# Did an Ultra-Low Emissions Policy on Coal-Fueled Thermal Power Reduce the Harmful Emissions? Evidence from Three Typical Air Pollutants Abatement in China

**DOI:** 10.3390/ijerph17228555

**Published:** 2020-11-18

**Authors:** Penghao Ye, Senmao Xia, Yu Xiong, Chaoyang Liu, Fei Li, Jiamin Liang, Huarong Zhang

**Affiliations:** 1 School of Business Administration, Zhongnan University of Economics and Law, Wuhan 430073, China; paulyph@whu.edu.cn (P.Y.); zhrzuel@126.com (H.Z.); 2 Newcastle Business School, Northumbria University, Newcastle upon Tyne NE1 8ST, UK; w15029125@northumbria.ac.uk; 3 International Centre for Transformational Entrepreneurship & Center for Business in Society, Coventry University, Coventry CV1 5FB, UK; ac5150@coventry.ac.uk; 4 Surrey Business School, University of Surrey, Surrey GU2 7XH, UK; 5 Research Center for Environment and Health, Zhongnan University of Economics and Law, Wuhan 430073, China; lcy@zuel.edu.cn (C.L.); lifei@zuel.edu.cn (F.L.)

**Keywords:** thermal power industry, emission reduction, ultra-low emission, SO_2_, NO_x_, smoke dust, difference-in-difference

## Abstract

Thermal power generation based on coal-fired power plants has the advantages of stability and controllability and has been the largest source of electricity supply in China. Coal-fired power plants, however, are also accompanied by high carbon emissions and the release of harmful substances (mainly including sulfur dioxide, nitrogen oxides, and smoke dust), and are even regarded as the “chief criminal” in terms of air pollution. However, thermal power is also a pioneering industry involved in several environmental regulations and cleaner production techniques before other industries. Evidence of this is China’s ultra-low emissions (ULE) policy on coal-fired power plants, implemented in 2015. To verify this policy’s effect, this study treats ULE as an exogenous impact variable, examining its emissions reduction effect on SO_2_, NO_x_, and smoke dust in Eastern and Central China using the difference-in-difference method (DID). The results show that the total emissions of the three pollutants were abated by 0.133%, 0.057% and 0.036% in Eastern, and by 0.120%, 0.035% and 0.043% in Central China at every 1% rise of thermal power generated after ULE. In addition, several other factors can also argue for the promotion of thermal power. Other industries, such as steel or chemical, have proven that they can contribute significant SO_2_ and NO_x_ emissions. Based on these results, we provide suggestions on synergistic emissions reduction among multiple industries, as well as a discussion on the necessity of implementing ULE in Western China.

## 1. Introduction

### 1.1. Development History of Thermal Power

**Figure 1:**
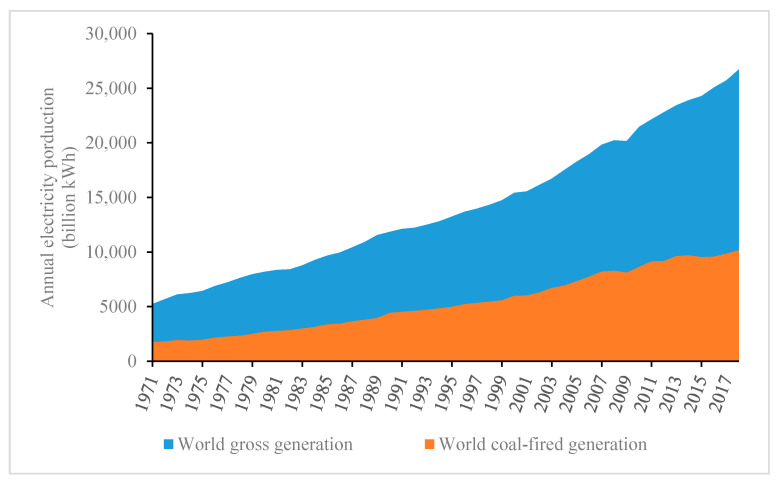
World gross generation and world coal-fired power generation from 1971 to 2018 (data source: World Bank/International Energy Agency).

Thermal power generation is a fundamental power source in modern countries. Due to the sufficient reserves of coal and the stable and controllable features of electricity generation, this means of electricity generation, mainly based on coal firing, has become the mainstay among most countries since the Second Industrial Revolution. With the rapid growth in total electric energy production, there has been a corresponding rapid increase in coal consumption. Figure 1 displays the world’s gross electricity generation and coal-fired electricity generation, where the coal-fired power generation in 2011 was 9.13 trillion kilowatt-hours (3.12 × 10^16^ Btu), accounting for 41.2% of the world’s gross generation that year, which was 22 trillion kilowatt-hours (7.51 × 10^16^ Btu). This statistical outcome indicates that thermal power, mainly based on coal-burning, remains an indispensable form of power generation.

**Figure 2:**
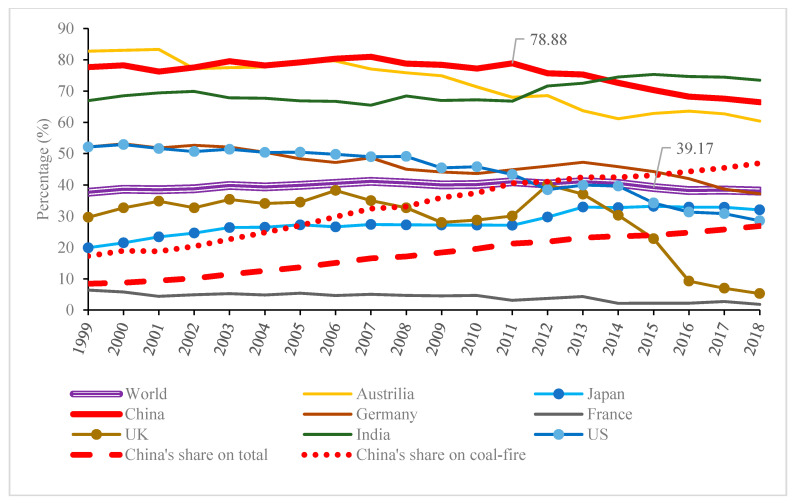
Proportion of coal-fired power generation to total power generation from 1999 to 2018 (data source: World Bank/International Energy Agency).

The statistical graph in Figure 1 also reveals that the proportion of coal-fired generation has grown rather than declined, from 30.5% in 1978 to 41.2% in 2011. Even this growing trend cannot be considered to have reversed since 1978, as coal-fired generation still accounted for 39.17% in 2015 (see the triple line in Figure 2). One significant factor that has led to these statistics is China, where the installed capacity for thermal power mainly depends on coal-fired power, and which has witnessed a massive boom after China’s reform and the enactment of the opening-up policy in 1978, leading to both the share of total electricity (the long dash line) and coal-fired electricity (the short dash line) in the world growing persistently. In fact, coal-fired power has long served as the backbone of the electric supply in China, whose proportion had remained at ~80% before 2011, which is twice the world average, as shown in Figure 2.

The rapidly growing installed capacity and generated energy from coal-fired power has yielded and will continue to yield a set of environmental issues. Apart from the emissions of CO_2_ and poisonous carbon monoxide gas (CO, a product of incomplete combustion), other substantial emissions, including sulfur dioxide (SO_2_), nitrogen oxide (NO_x_), and smoke dust (soot dust), are also products of coal burning, as coal contains N and S, along with other impurities [1].

Due to these social complaints, numerous countries have set regulations and technical routes to limit and regulate thermal power. The ultra-low emissions (ULE) policy is China’s attempt to deal with pollutant discharge, mainly focusing on coal-fired power plants as they are the main thermal source in China. However, due to the appeal of thermal power, certain industries that can also release similar pollutants may have “escaped” the public eye, with the establishment of fewer environmental regulations for these industries (discussed in the following sections).

### 1.2. History of Thermal Power Emissions Reductions in China

**Figure 3:**
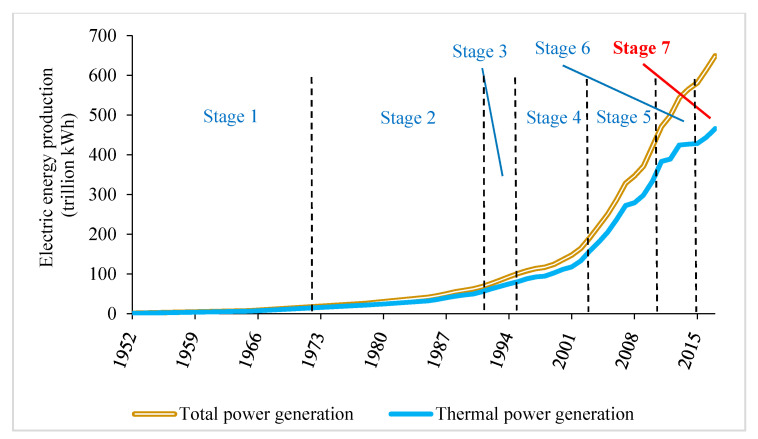
Statistics on China’s electricity production at different stages from 1952 to 2017 (data source: National Bureau of Statistics of China).

Air pollution problems have been a global concern since the beginning of the 21st century. During the 10 years after China’s admittance to the World Trade Organization (WTO), the domestic installed capacity and annual electricity production increased rapidly, along with its booming economy, as shown in Figure 3. However, after 2010, the frequent occurrence of hazy conditions in multiple regions of China has incited widespread social concern [2]. One typical event was a nationwide occurrence of haze in January 2013, which caused an increase in traffic accidents and medical diagnoses, leading to a loss of 2.3 billion RMB [3]. Particularly in Northern China, these smoggy haze conditions occur more frequently because, apart from coal-fired power, there is relatively less precipitation in this region, as well as increased coal power generation for central heating from 4 to 6 months annually [4]. This pollution problem also largely exists in Eastern China, where the economy is larger than Central and Western China, such that there are increased energy requirements.

Since 1991, China has gradually implemented national standards, upgraded its technologies, and promoted legislation to enhance emission limits with respect to thermal power plants. Based on the promulgation of national standards and legislation, the effort to reduce pollution from coal-fired power plants can be divided into seven stages [5], as labelled in Figure 3. Stage 1 existed before 1972, where no substantial emissions restriction policy was in place during that period. In Stage 2, which began in 1973 and lasted 18 years, the government implemented a loose emissions restriction policy, which only imposed restrictions on the emissions rate and height for thermal power plants, but did not set any restrictions on the emissions load. A substantial restriction policy was established in Stage 3 (1991–1995), i.e., the emission standard of air pollutants for thermal power plants [6], requiring the installation of dust removal facilities to ensure that the concentration of smoke dust was not more than 600 mg/m^3^. Based on this first significant national standard, a more complete combined emissions limitation policy [7] was formulated in Stage 4 (1996–2003), which established emissions limits for SO_2_, NO_x_, and smoke dust, of 1200, 650, and 200 mg/m^3^, respectively. The limits on these three air pollutants were successively strengthened during the next two stages [8,9], not exceeding 100, 100, and 30 mg/m^3^, respectively, throughout the entire country, along with limits on SO_2_ and smoke dust emissions of 50 and 20 mg/m^3^ in key regions, which were demarcated in Stage 6.

After implementing the emission standard of air pollutants for thermal power plants [9], China has continued to explore emissions reduction measures in the thermal power industry. In 2015, a nationwide government document entitled “Full implementation of the ultra-low emission and energy-saving transformation work plan for coal-fired power plants” [10] was released (hereinafter referred to as “ultra-low emissions” or “ULE”). The release of the ULE indicates the official initiation of the current stage (7), which has been the most vigorous implementation stage in China to date, setting the most stringent emissions reduction requirements.

### 1.3. Policy Release Background

Before the formal issue of the ultra-low emissions policy, China had completed a significant amount of investigations on the current status of thermal power emissions in various regions, as well as summarizing the effects that previous regulation policies have had on the thermal power industry. Table 1 lists the emission standards of great significance implemented in the past decade.

In 2014, the year before the release of the ULE, two national-level documents were enacted, serving as forerunners to the ULE. The first document was the Energy Development Strategy Action Plan (2014–2020) [11], which established the future path(s) of the Chinese energy supply, proposing that domestic fossil energy reserves should be the foundation of China’s long-term energy sources for reasons of security and reliability. The second document, entitled “Coal-fired energy-saving emission reduction upgrade and transformation action plan” (2014–2020) [12], was jointly issued by three key national government departments, establishing a specific, strict emissions standard for the coal-fired power plants in China. This standard considered the different economic strengths of different regions in China by dividing the country into three regions, i.e., Eastern China, Central China, and Western China, and setting stringent emissions restrictions for SO_2_, NO_x_, and smoke dust, not to exceed 35, 50, and 10 mg/m^3^, respectively (under an oxygen content of 6%). Under this standard, Eastern and Central China should finally reach these limits, but the latter may be given a longer time allowance considering the differing economic positions among these regions. For Western China, whose economy is the least developed, this standard is a recommendation rather than an obligation.

The technological route to reduce pollutant emissions had not yet been determined before 2015, despite the previous standards that clearly established the emissions limits for coal-fired power plants. The “Full implementation of the ultra-low emission and energy-saving transformation work plan for coal-fired power plants (ULE)” [10] policy was formally introduced at the end of 2015. This ULE policy stressed that all coal-fired power plants with reconstruction conditions in China should achieve the ultra-low emissions standards by 2020. The only differences were the deadlines for Eastern, Central, and Western China, which were 2017, 2018, and 2020, respectively.

### 1.4. Policy Implications for China

**Figure 4:**
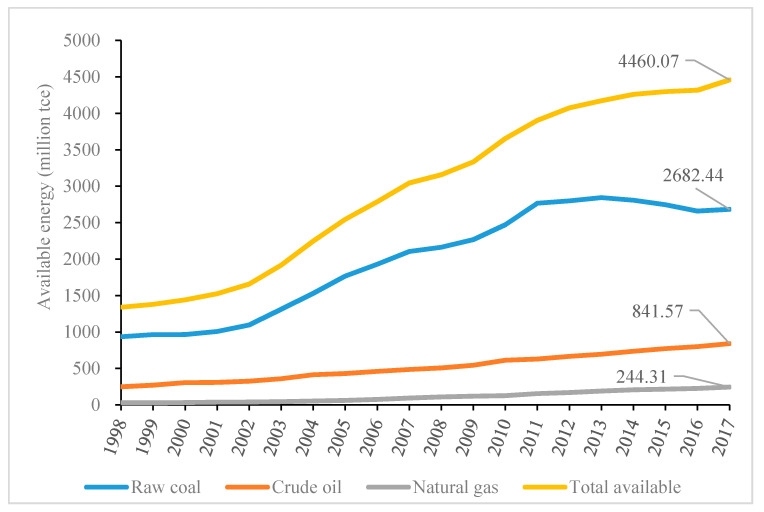
The availabilities of three fossil fuels and their total amount in China from 1998 to 2017 (Data source: China Energy Statistical Yearbook).

Coal has served as the backbone of China’s energy source for a long time [13], accounting for around 70% of the total gross electricity production. In terms of a unified measurement, i.e., tons of standard coal (tce), the available raw coal in 2017 was 2682.44 million tce, whereas that of crude oil and natural gas was 841.57 and 244.31 million tce, respectively, as shown in Figure 4. Moreover, compared with other forms of power generation, the dominant position of coal-fired power is consistent with China’s national conditions, i.e., other energy sources cannot entirely fulfill the energy demands of China. For hydropower, the dry season may result in power supply shortages if no other forms of energy can compensate [14], and may also offer finite contributions to heating in winter for Northern China, provided by coal firing. Renewable energies, such as solar and wind, cannot serve as stable sources [15], and may also have their own environmental issues, such as marine acoustic effects that may affect marine organisms [16]. The widespread “not-in-my-backyard” mindset in China with respect to nuclear power will restrict its expansion into inland areas [17].

Compared with fuel oil and natural gas, combustion coal normally contains more non-hydrocarbon impurities [18]. In addition to 2.46 tons of CO_2_, the untreated combustion of 1 tce coal produces 12–15 kg of SO_2_, 16–20 kg of NO_x_, and 50–70 kg of smoke, among which the emissions of SO_2_ and smoke are significantly larger than that of crude oil and natural gas. For this situation, the significance of the ultra-low emissions policy is evident because, with the adoption of ULE technology, the removal efficiency of SO_2_, NO_x_, and dust can exceed 95%, 70%, and 99.8%, respectively. Other noxious pollutants, such as SO_3_ or mercury compounds, can also be filtered out, achieving the goal of “zero-emissions” of pollutants [10]. Consequently, the ULE may play a vital role in building an environmentally friendly and sustainable atmospheric environment in coal-fired based countries, such as China.

### 1.5. ULE Technical Route

**Figure 5:**
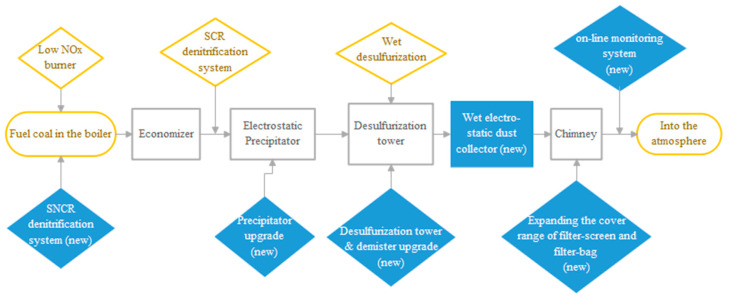
Flowchart of ULE implementation.

There are a variety of pathways to achieve ultra-low SO_2_, NO_x_, and smoke dust emissions. However, most are implemented by installing new equipment for denitrification (SO_2_ reduction), desulfurization (NO_x_ reduction) [19], and dedusting (smoke reduction) [20], or by enlarging the capacity of existing purification equipment [21]. Further, dust can be reduced by switching to cleaner storage and transportation processes for coal [22,23]. These measures have been generally adopted in Eastern and Central China after the implementation of the ULE policy. Figure 5 depicts a typical ULE realization scheme for existing coal-fired power plants.

As shown in Figure 5, for an existing power plant, the measures in the diamond-shaped non-transparent blocks are the key flow paths that will reduce the emissions of the three pollutants. One Chinese thermal power generation group, Shenhua, invested 240.45 million CNY to retrofit four coal-fired units in a power plant, successfully cutting down the concentrations of SO_2_, NO_x_, and dust by 47.5%, 91.6%, and 78.6%, respectively [24].

## 2. Literature Review and Hypothesis

### 2.1. Background on ULE Policy in the Power Industry

Since 2015, the quantification of emissions reduction in China’s thermal power industry has been unprecedented, where the ULE policy has received a high response from most of the power generation groups in China, and has attracted the attention of a number of researchers. Han [25] pointed out that the ULE is an applicable mitigation option in a country where coal power accounts for more than 50% of the power generation, and the mitigation effect can be enhanced using ultra supercritical (USC) technologies. A case study of 118 coal-fired power plants in Beijing, Tianjin, and Hebei Province showed that the ULE can promote a removal rate of 60%, 11%, and 29% for SO_2_, NO_x_, and dust, respectively. Zhao and Zhu [26] detected the changes in PM_2.5_ in Jiangsu Province, an eastern province where both the production and the proportion of thermal power have ranked in the top three highest in China in the past decade. They observed that the annual average mass concentration of PM_2.5_ decreased by 42.5% from 2013 to 2017, with an evident contribution from the ULE. Tang et al. [27] performed an ex-post study using a continuous monitoring systems network to track the emissions from 96–98% of the thermal power plants in China, finding that the emissions of SO_2_, NO_x_, and particulate matter declined by 65%, 60%, and 72%, respectively, from 2014 to 2017. Jin et al. [28] mentioned that China’s ULE reconstruction spent 0.07 to 0.37 USD per kWh and resulted in a higher feed-in tariff, but is still cost-effective compared to gas power. Wen et al. emphasized that ULE can also lessen the mercury emission in coal-fired power plants [29].

These studies have all shown that the ULE has been effective to varying degrees. However, most studies have focused on the emissions of power plants themselves, where the overall emissions, including other possible sources, have not yet been considered. Despite testing the abatement of thermal power, we cannot simply ignore other industries with no reduction policies. Another deficiency of most of the previous ULE literature is that their third tested emission is PM_2.5_, which differs from what the Chinese official document [10] has required, i.e., smoke dust. Moreover, a long-term dataset can provide a more explicit contrast between before and after the implementation of the ULE. Based on this, we selected data from the officially published statistical yearbook from 2011–2017 to analyze these effects [30,31,32], and considered other potential sources of these pollutants.

### 2.2. Review on Policy Testing Methods

First proposed by Ashenfelter [33], difference-in-difference (DID) is a special method to evaluate the effects of pollution by estimating the training programs on economic growth, unemployment reductions, and poverty alleviation. The theoretical basis of DID is the premise that a natural or a quasi-experiment, i.e., an experiment (e.g., an exogenous policy) affected by an experimental group (or treated group), is selected for comparison with an unaffected matched group (or control group), which had similar variation trends with the treated group before the experiment [34]. If the treated group yields a significant difference from the control group after the experiment, and other irrelevant factors have been eliminated, then this natural or quasi-experiment is shown to be significantly effective [35].

This method of estimation has expanded into the environmental field. Greenstone and Hanna [36] evaluated the introduction of environmental regulations in India by examining air quality issues and the water environment, concluding that the 1981 Air Act has been effective, but the 1974 Water Act has had relatively few positive results. Yang et al. [37] employed the Propensity Score Matching and DID (PSM-DID) method to verify the effect that the construction of a high-speed railway has on environmental pollution, showing that there has been a significant 7.35% reduction in the pollution. As ULE conforms to an exogenous policy exerted to control emissions, we used the DID approach to estimate the abatement results of SO_2_, NO_x_, and smoke dust in China.

### 2.3. Research Hypothesis

As previously mentioned, the overall emissions of SO_2_, NO_x_, and smoke dust are not only from the power industry, but the power industry has generated a significant amount of these emissions. We must identify whether the implementation of ULE in the power industry alone can significantly reduce the emissions of SO_2_, NO_x_, and smoke dust in the overall atmosphere in China. Hence, we propose the following hypothesis (H1):

**Hypothesis 1 (H1).**  *The ultra-low emission policy, specific to coal-fired power stations in China, can significantly reduce the emissions of SO_2_, NO_x_, and smoke dust in the entire country.*

Moreover, the thermal power industry alone does not contribute to the existence of SO_2_, NO_x_, and smoke dust. Other industries, such as the iron, steel, and chemical industries, can also release such pollutants into the atmosphere. Meanwhile, traffic emissions also contain considerable amounts of nitrogen oxide and particulate matter. The emissions from these industrial practices and transportation cannot be neglected, despite the fact that the thermal power industry has been the major focus of criticism with respect to air pollution. Based on the fact that there have been relatively few similar emissions reduction policies for industries other than thermal power, we propose a second hypothesis (H2):

**Hypothesis 2 (H2).**  *Other industries, aside from the thermal power industry, did not noticeably contribute to the emissions of SO_2_, NO_x_, or smoke dust during the ULE implementation period.*

## 3. Methods

### 3.1. Selection of the Treated and Control Groups

**Figure 6:**
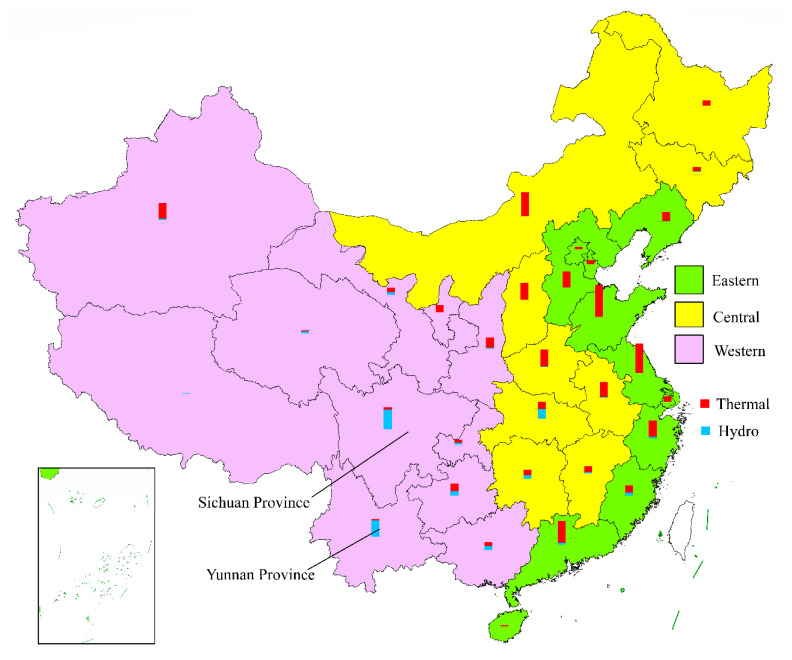
Distribution of thermal and hydropower generation in Eastern (green), Central (yellow), and Western (purple) China in 2017 (data source: [31]).

In this study, we selected Eastern and Western China as the treated group to examine whether the ULE policy has achieved its goal of emissions reduction. We selected Western China as a reasonable control group for comparison. We used Eastern and Central China as the experimental groups due to the following reasons: in combination, the two regions shared (1) 72% of the population and 82.6% of the GDP in 2019 and (2) approximately 80%/83% of the total/thermal power generation in the past 10 years in mainland China. Figure 6 depicts the partitioning of Eastern, Central, and Western China, as well as labeling of the comparison of the thermal and hydropower generation in these three regions (data from 2017) [31]. This map indicates that thermal power, mainly based on coal-firing, dominates the power supply chain in Eastern and Central China, but is significantly less than hydropower in Western China. Moreover, most environmental issues have originated from these two regions, which urgently require relevant treatment policies such as the ULE.

**Figure 7:**
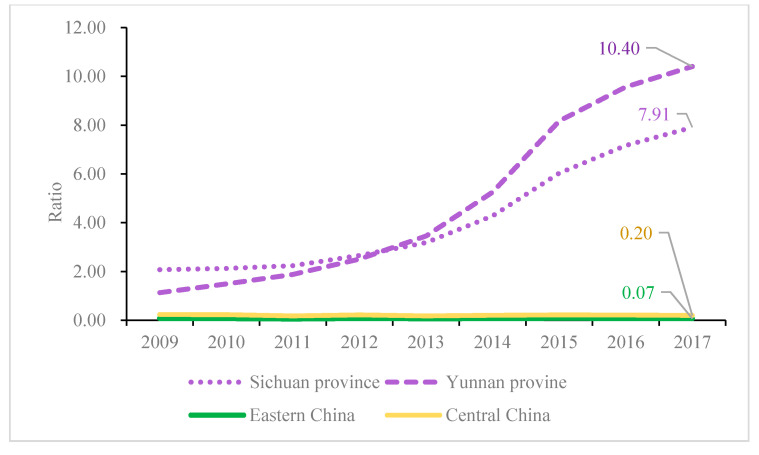
Proportion of electricity generated by hydropower/thermal power from 2009 to 2017 in Eastern China, Central China, and in two western provinces.

Western China has not been treated with relevant policies due to the following reasons. First, although the implementation scope of the ULE policy concerns the entirety of mainland China, the timetables for the different areas to implement this policy are not the same. As previously mentioned, the timeframe for Western China to attain the objectives of the ULE is 2020, whose wording is “strive for” rather than “must.” In contrast, Eastern and Central China, which contain most of the economy and population, have much stricter requirements with respect to both the timeframe and target. A second reason is that the energy structure in Eastern and Central China are similar, where both rely heavily on thermal power based on coal. In contrast, Western China has abundant hydropower resources, especially in the Sichuan and Yunnan provinces. Figure 6 is an ArcGIS map that shows the power distribution in China, where we can observe a contrast between Eastern/Central China and Western China in terms of the generation of thermal and hydropower. Considering that Western China occupies 56.4% of China’s land area, both the share of the total power generation and the share of thermal power generation in the Western region is comparatively low, only occupying ~20% of the total and 16% of the thermal generation in China in the past 10 years. Certain western provinces rely heavily on hydroelectric power generation rather than fossil energies. Figure 7 shows the annual electricity generated by two typical western provinces, i.e., Sichuan and Yunnan provinces, along with a comparison to Eastern and Central China.

Based on Figure 7, it is easy to observe that the hydro/thermal power generation ratio in Eastern and Central China have remained at approximately 0.07 and 0.20, respectively, from 2009 to 2017, whereas this ratio in Sichuan and Yunnan provinces has been larger than one since 2009, and has even grown exponentially since then, reaching 7.91 and 10.40, respectively, in 2017. This shows that these two western provinces do not rely on thermal power. Due to the considerably low percentage of thermal power generation and the relatively loose implementation of the ULE in Western China before 2017, these two western provinces can be deemed as having no ULE policy implemented before 2018, i.e., they can be classified as the control group.

### 3.2. Model Specification

To test the ULE policy’s effectiveness from 2015 to 2017, difference-in-difference models, with a combination of thermal power generation [38], were set as follows:

(1) $$\ln SO_{2}{}_{it}=\beta_{0}+\beta_{1}{ULE}_{i}\times {year}_{t}\times lnthermal+\underset{j}{\sum }\beta_{j}\times {Control}_{jt}+\delta_{i}+\mu_{t}$$

(2) $$\ln{\left(\frac{{SO}_{2}}{thermal}\right)}_{it}=\beta_{0}+\beta_{1}{ULE}_{i}\times {year}_{t}\times lnthermal+\underset{j}{\sum }\beta_{j}\times {Control}_{jt}+\delta_{i}+\mu_{t}$$

(3) $$\ln{NO}_{xit}=\beta_{0}+\beta_{1}{ULE}_{i}\times {year}_{t}\times lnthermal+\underset{j}{\sum }\beta_{j}\times {Control}_{jt}+\delta_{i}+\mu_{t}$$

(4) $$\ln{\left(\frac{NO_{x}}{thermal}\right)}_{it}=\beta_{0}+\beta_{1}{ULE}_{i}\times {year}_{t}\times lnthermal+\underset{j}{\sum }\beta_{j}\times {Control}_{jt}+\delta_{i}+\mu_{t}$$

(5) $$\ln{Dust}_{it}=\beta_{0}+\beta_{1}{ULE}_{i}\times {year}_{t}\times lnthermal+\underset{j}{\sum }\beta_{j}\times {Control}_{jt}+\delta_{i}+\mu_{t}$$

(6) $$\ln{\left(\frac{Dust}{thermal}\right)}_{it}=\beta_{0}+\beta_{1}{ULE}_{i}\times {year}_{t}\times lnthermal+\underset{j}{\sum }\beta_{j}\times {Control}_{jt}+\delta_{i}+\mu_{t}$$

(7) $${DID}_{it}={ULE}_{i}\times {year}_{t}\times lnthermal$$

In models (1), (3), and (5), thermal represents thermal power generation (unit: billion kWh), where lnthermal is its common logarithm values of the three emissions, i.e., SO_2_, NO_x_, and smoke dust (unit: million tons). The subscripts *i* and *t* represent the region and year, respectively. ${ULE}_{i}$ and ${year}_{t}$ are two dummy variables, where ULE is the policy dummy and year is the time dummy. If *i* is “East” or “Central,” which indicates that the region has implemented the policy, then ${ULE}_{i}$ is equal to 1; otherwise, when *i* is “Yunnan” or “Sichuan,” then ${ULE}_{i}$ is equal to 0, indicating that the region has not implemented the policy. Likewise, if *t* ≥ 2015, then ${year}_{t}$ = 1, which indicates that this year is the year after ULE execution; otherwise, if *t* < 2015, then ${year}_{t}$ = 0. Formula (7) provides the policy effect, $DID_{it}$, which indicates the emissions reduction effect from the ULE policy.

To examine the emissions reduction in terms of the average thermal power generation, we used models (2), (4), and (6), where ${\left(\frac{SO_{2}}{thermal}\right)}_{it}$, ${\left(\frac{NO_{x}}{thermal}\right)}_{it}$, and ${\left(\frac{Dust}{thermal}\right)}_{it}$ represent the emissions of SO_2_, NO_x_, and smoke dust, respectively, from one unit thermal power generation (unit: million tons/kWh). We used ${Control}_{jt}$ to signify the other industrial products that can also generate these three pollutants (see Table 2). Finally, $\delta_{i}$ represents the individual fixed effects and $\mu_{t}$ is the deviation.

### 3.3. Data Sources and Variable Descriptive Statistics

All of the thermal power generation panel data at the provincial level were collected from the China Energy Statistical Yearbook [31], all of the emissions data for SO_2_, NO_x_, and smoke dust were collected from the China Statistical Yearbook on Environment [32], and other data for the control variables derive from the China Industry Statistical Yearbook and Finance Yearbook of China [39]. Table 3 provides the descriptive statistics of the explained variables and main explaining variables.

## 4. Empirical Results

### 4.1. Effect Test of ULE

#### 4.1.1. Emissions Reduction for SO_2_

To inspect the emissions reduction outcome for SO_2_ in Eastern and Central China, we applied DID models (1.1a) and (1.1b) for regression analysis, as listed in Table 4. The results show that the ULE policy on SO_2_ emissions is fairly effective, as the coefficients of the *DID* model are both statistically significant at the 1% level. After the implementation of the ULE policy, a 1% increase in the thermal power generation will reversely reduce the overall SO_2_ emissions by 0.133% and 0.120%, respectively, in the Eastern and Central Provinces, or yield a reduction of 0.149% and 0.144% in terms of the average SO_2_ emissions (megaton/billion kWh). Therefore, for SO_2_ alone, Hypothesis 1 (H1) is valid in both Eastern and Central China, as well as being both valid for the overall SO_2_ and unit SO_2_ emissions reduction.

To obtain the validity of Hypothesis 2 (H2), we used the significance of two control variables. The coefficients in rows 3 and 5 and columns 5 and 6 of Table 4 display the statistical significance of the two control variables related to SO_2_, where we observe that crude steel and sulfuric acid can significantly contribute to SO_2_ emissions in Eastern China, whereas this contribution is non-significant in Central China. In Eastern China, a 1% increase in the production of crude steel and sulfuric acid would yield a 0.517% and 0.321% increase, respectively, in the SO_2_ emissions. This indicates that elevated SO_2_ emissions cannot be attributed to the thermal power industry alone; other secondary industries, such as the iron, steel, or chemical engineering industries, can also discharge a substantial amount of SO_2_. Despite this, there have been relatively few relevant emissions reduction policies for these industries implemented before 2017, other than thermal power. Based on this, H2 is not valid for the entirety of China, such that restrictions on the thermal power industry alone may not be sufficient to reduce the overall SO_2_ emissions. When the explained variable is $\ln\left(\frac{SO_{2}}{thermal}\right)$, $\ln\left(\frac{NO_{x}}{thermal}\right)$, or $\ln\left(\frac{Dust}{thermal}\right)$, there is little meaning when counting the other industries as control variables, because no similar emissions data sharing have been considered. Hence, we do not discuss H2 for these cases.

#### 4.1.2. Emission Reductions for NO_x_

To inspect the emissions reduction outcomes for NO_x_ in Eastern and Central China, Table 5 lists the regression results from models (1.2a) and (1.2b) using DID estimation. The results show that the ULE policy also played an integral role in NO_x_ emissions in Eastern and Central China, as all of the coefficients in column 3 are statistically significant at the 1% level. After ULE execution, a 1% rise in thermal power generation would not increase, but rather reduce NO_x_ emissions, i.e., by 0.057% in Eastern China and 0.053% in Central China. Meanwhile, the unit NO_x_ emissions, ($\frac{NO_{x}}{thermal}$), can be reduced by 0.065% and 0.098% in Eastern and China, respectively. Hence, H1 is also valid in terms of the NO_x_ emissions reduction, such that the ULE policy is also effective at NO_x_ abatement.

The yield of chemical fertilizer (in NPK) and vehicle holding served as the control variables in models (1.2a) and (1.2b), where the contribution of fertilizer to NO_x_ emissions is non-negligible in both Eastern and Central China, with a 1% significance level in Eastern China and 10% level in Central China. This indicates that the production process of nitrogen-containing fertilizers is another major source of NO_x_ emissions; however, the Chinese government has currently not implemented any emission policies similar to the thermal power policies around 2015. Consequently, H2 is not valid regarding NO_x_ emissions.

For other control variables, however, vehicle holding did not play an important role in Eastern China, and even negatively affected the NO_x_ emissions in Central China, as listed in rows 3 and 5 and column 6 of Table 5. This indicates that vehicle exhaust may not be a significant nitrogen oxide source in Eastern and Central China, which may be explained by the progressively stricter emissions standards for cars in China. This outcome stresses the importance of collaborative reductions among different industries.

#### 4.1.3. Emissions Reduction for Smoke Dust

Table 6 lists the regression analysis results for smoke dust emissions reduction using the DID approach. All of the DID coefficients are statistically significant at a 1% level, except for one value in row 3 in the Eastern region, proving the significant effects of the ULE policy in terms of the reduction, similar to the other pollutants. The ULE has yielded an abatement of the overall smoke dust of 0.036% in the Eastern provinces and 0.043% in the Central provinces with a 1% increase in generation since 2015, which is a 0.052% and 0.041% reduction in the unit smoke dust emissions in terms of thermal power generation. Therefore, *H_1_* remains valid when the explained variable is smoke dust.

We then considered two possible control variables, i.e., vehicle holdings and fixed investments, which can also generate smoke dust when operating a vehicle or the construction of infrastructure. In this case, unlike the two previous cases, these two factors do not have any pronounced contribution to the emissions of smoke dust, as none of their coefficients were statistically significant in Table 6. A possible explanation for this weak link between the emissions of smoke dust and vehicle operation or the fixed assets of construction is that the national and local governments in China had already attached more importance to the municipal environment before the implementation of the ULE, with the intensive treatment of smoke and dust. The measures to mitigate municipal pollutants like smoke dust include the use of more road sprinklers, distributing subsidies to the buyers of clean-energy vehicles, or strengthening the supervision of illegal pollutant discharge [40]. This result may also reflect collaborative efforts to reduce smoke dust, which have been more effective than that for SO_2_ or NO_x_. A wide range of cross-industry countermeasures have already been established, in addition to the ULE [41]. Consequently, H2 is valid when the explained variable is smoke dust.

### 4.2. Robustness Test

#### 4.2.1. Parallel Trend Test

**Figure 8:**
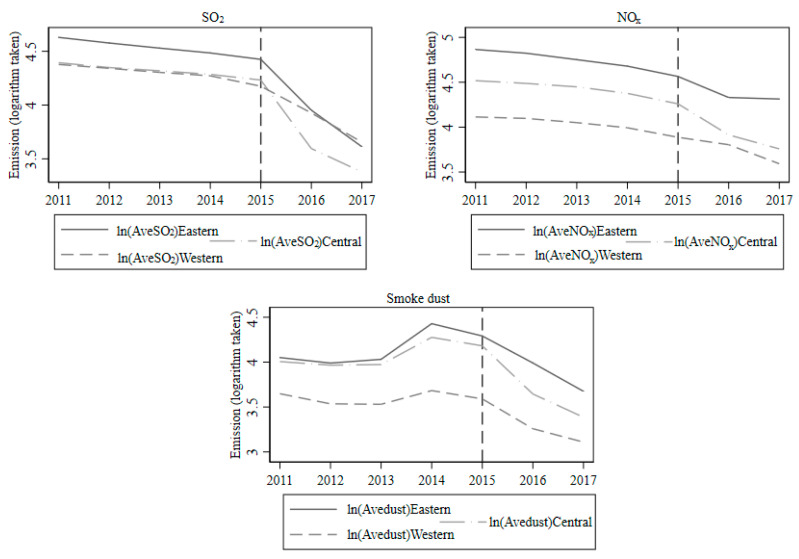
Parallel trend test for the treated and control groups.

One requisite precondition of employing the DID method is that, before the implementation of the policy, the treatment and control groups have similar variation trends, where significant differences only occur after implementation [35]. Therefore, to ensure that this model matches this precondition, we must verify whether the provinces in Eastern and Central China (treated groups) have similar emissions trends with the two eastern provinces (control group). Figure 8 plots the emissions trends for the treated and control groups, where the emissions of SO_2_, NO_x_, and smoke dust were all averaged as a unit province. Each region’s unit provincial value is averaged based on the summation of all the logarithmic values of the provinces and municipalities. As the municipalities do not belong to any province in China, we then treated each municipality as 0.3 of the province in the calculations.

Based on Figure 8, the eastern region in China has been the main producer of the average SO_2_ and NO_x_ emissions, and significantly outweighs the two western provinces in terms of smoke dust emissions, along with the central region. However, despite the different emissions load, we can observe that the treated groups and the control group do have approximately the same trend with an almost steady difference before 2015, which is the year of ULE implementation. Consequently, the parallel trend for the three groups supports the use of the DID method.

#### 4.2.2. Placebo Test

We have confirmed that the ULE policy reduces the emissions from thermal power plants, but certain other environmental protection policies may have also affected the discharge of SO_2_, NO_x_, and smoke dust before 2017. Therefore, to ensure that these effects derive from the ULE rather than any other policies, which may contribute to the emissions reduction, a placebo test using a counterfactual analysis is necessary. At this point, we moved the ULE implementation year forward by two to four years, followed by an examination of the statistical significance, as listed in Table 7. Only when the DID coefficients are less significant or non-significant can the placebo test pass, revealing that the ULE policy alone is effective.

Based on Table 7, we can observe that, when we set the ULE execution year to before 2015, the coefficients of the policy effects are no longer statistically significant when the explained variable is NO_x_ or smoke dust, which fully supports the unique effect of the real ULE policy executed in 2015. When the explained variable is SO_2_, the DID coefficient is significant at the 10% level. The reason for this significance may be that certain environmental policies had already been implemented before 2015, such as the “Emission standards for air pollutants from coal-fired power plants” in 2011 (see Table 1), which placed loose restrictions on coal-fired power plants. Moreover, the effect of emissions reduction is only 0.019% and 0.015% in Eastern and Central China, respectively, which is less than the actual ULE, i.e., 0.133% and 0.120%, respectively (Table 4). Hence, we can assume that the results of the DID test for the ULE are robust, and that ULE has played a significant role in the abatement of SO_2_, NO_x_, and smoke dust in Eastern and Central China, where coal-fired power has been the largest long-term energy source.

## 5. Conclusions and Suggestions

In this study, we tested the ULE policy using the DID method, first proposing the hypothesis that the ULE can significantly reduce the emissions of SO_2_, NO_x_, and smoke dust (H1). Our results show that H1 is valid, as the introduction of the ULE has substantially reduced these three air pollutants in Eastern and Central China, where thermal power accounts for more than 78% and 75% of the total gross generation, respectively, i.e., about 48% and 34% of the entire country’s gross generation. Since 2015 when ULE was implemented, each 1% incremental thermal power generation can reversely reduce the total emissions of SO_2_, NO_x_, and smoke dust by 0.133%, 0.057% and 0.035%, respectively, in Eastern China, along with 0.120%, 0.053% and 0.043%, respectively, in Central China. Western China was not tested in this paper due to its low percentage of thermal/gross energy production and relatively loose ULE requirements. Future studies may examine particular western provinces when the ULE policy becomes fully effective there.

Aside from the successful emissions reductions in the thermal power industry, this paper also justified that thermal power is not the only distinct source of pollutants. A second hypothesis (H2) was raised to verify whether some other industries can also affect emissions. The results largely support our argument, as we found that the steel and chemical industries are highly correlated with SO_2_ in Eastern China while the fertilizer industry contributes significantly to NO_x_ emissions in Eastern and Central China. The only positive outcome is for smoke dust, which has not been affected by other possible sources such as vehicle operation and capital constructions, possibly because of the collaborative reduction.

Based on these results, solely focusing on the power industry cannot thoroughly mitigate air contamination in a country that contains numerous secondary industries. The public should also focus on the supervision of other polluting industries. The mitigation of smoke and dust has proven that synergetic abatement is feasible. However, a wider range of reduction policies applied to multiple industries, rather than a single industry, remains an urgent need, such as prioritizing alternative energies or controlling farm emissions [42]. Collaborative reductions have captured the attention of the government. For example, an official publication, entitled “Suggestions on promoting the implementation of ultra-low emissions in the steel industry” [43], was recently proposed by the Ministry of Ecology and Environment of China, aimed at transforming 80% of the steel plants into low emitters of SO_2_, NO_x_, and particulate matter, referring to what the thermal power industry has achieved.

## Figures and Tables

**Table 1:** Current limitation standards and the limiting value of thermal power in China.

Emission Standard’s Name	Date of Issue	Concentration Limits (mg/m^3^)		
		SO_2_	NO_x_	Smoke Dust
Emission standards for air pollutants from coal-fired power plants	Sep 2011	100	100	30
Standards for the discharge of atmospheric pollutants from boilers ^1^	May 2014	300 (400)	300 (400)	50 (80)
Energy Development Strategic Action Plan (2014–2020) ^2^	June 2014	No new restricted limits proposed		
Coal-fired energy-saving emission reduction upgrade and transformation action plan (2014–2020)	Sep 2014	35	50	10
Full implementation of the ultra-low emission and energy-saving transformation work plan for coal-fired power plants ^2^	Mar 2015	35	50	10

Note: ^1^ Values without brackets are the emissions limits for newly-built boilers while bracketed values are the emissions limits for existing boilers. This standard is relevant for all industries, including coal-fired power plants using coal-fired boilers. The development level varies in different industries, such that the standard limits are relatively loose. ^2^ The Energy Development Strategic Action Plan (2014–2020) states that the emissions reduction limits for Eastern, Central, and Western China are different from the concentration limits, whereas the full implementation of the ultra-low emission and energy-saving transformation work plan for coal-fired power plant requires identical emissions reduction limits for Eastern, Central, and Western China, but with different completion times.

**Table 2:** Control variable set corresponding to the three emissions.

Name of Control Variable (Unit)	Symbol	Generated Pollutant	Taking the Logarithm or Not
Crude steel (million tons)	*steel*	SO_2_	Yes
Sulfuric acid (in 100%) (million tons)	*sulacid*	SO_2_	Yes
Chemical fertilizer (in NPK ^1^) (million tons)	*fertilizer*	NO_x_	Yes
Vehicle holdings (million)	*car*	NO_x_ and smoke dust	Yes
Annual fixed-asset investment (trillion CNY)	*fixedasset*	Smoke dust	Yes

Note: ^1^ “in NPK” means the weight of the chemical fertilizer has been converted into the content of nitrogen, phosphorus pentoxide, and potassium chloride [39].

**Table 3:** Descriptive statistics of the main variables.

Variable	Region	Obs	Mean	St.d	Min	Median	Max	Unit
*ULE*	East	63	1	0	1	1	1	-
	Central	70	1	0	1	1	1	
	West	14	0	0	0	0	0	
*Thermal*	East	63	220.279	129.192	58.732	225.860	514.288	billion kWh
	Central	70	147.094	95.242	51.625	89.721	375.033	
	West	14	44.850	13.520	23.804	46.072	62.810	
*SO_2_*	East	63	0.668	0.471	0.019	0.593	1.827	million tons
	Central	70	0.630	0.370	0.166	0.525	1.409	
	West	14	0.652	0.164	0.384	0.668	0.902	
*NO_x_*	East	63	0.862	0.499	0.142	0.843	1.801	million tons
	Central	70	0.728	0.355	0.255	0.606	1.665	
	West	14	0.330	0.071	0.224	0.340	0.429	
*Dust*	East	63	0.505	0.417	0.047	0.354	1.798	million tons
	Central	70	0.523	0.282	0.188	0.447	1.507	
	West	14	0.330	0.071	0.224	0.340	0.429	
DID=ULE×year×lnthermal	East	63	2.238	2.645	0	0	6.243	-
	Central	70	2.062	2.436	0	0	5.927	
	West	14	0	0	0	0	0	

Note: As certain control variables are missing in the data source, the eastern municipality of Beijing and the eastern Hainan province were not be incorporated in this empirical analysis.

**Table 4:** Regression outcome for the ULE’s reduction effect on SO_2_.

Explained Variable	Region	*DID*	Control Variable	lnsteel	lnsulacid	Constant	R^2^ within	Obs.
$\ln SO_{2}$	Eastern	−0.124 *** (−7.23)	No	-	-	−0.455 * (−1.87)	0.447	77
		−0.133 *** (−7.34)	Yes	0.517 *** (4.79)	0.321 *** (4.46)	−2.408 * (−6.39)	0.422	77
	Central	−0.121 *** (−8.84)	No	-	-	−0.388 *** (3.13)	0.529	84
		−0.120 *** (−8.56)	Yes	0.054 (0.29)	−0.039 (−0.54)	−518 (−0.96)	0.527	84
$\ln\left(\frac{SO_{2}}{thermal}\right)$	Eastern	−0.140 *** (−7.68)	No	-	-	−5.365 *** (−32.51)	0.483	77
		−0.149 *** (−7.59)	Yes	0.057 (0.45)	0.299 *** (3.58)	−5.724 *** (−13.02)	0.477	77
	Central	−0.148 *** (−8.55)	No	-	-	−4.960 *** (−60.11)	0.531	84
		−0.144 *** (−8.74)	Yes	−0.420 *** (−2.69)	0.109 ** (1.98)	−3.824 (−8.63)	0.506	84

Note: ***, **, and * represent the statistical significance at the 1%, 5%, and 10% level. The values in the brackets are the t statistics.

**Table 5:** Regression outcome for the ULE reduction effect on NO_x._

Explained Variable	Region	*DID*	Control Variable	lnfertilizer	lncar	Constant	R^2^ within	Obs.
$\ln{NO}_{x}$	Eastern	−0.078 *** (−9.37)	No	-	-	−0.273 (−1.50)	0.587	77
		−0.057 *** (−4.29)	Yes	0.213 *** (3.70)	−0.167 (−1.51)	0.053 (0.24)	0.646	77
	Central	−0.093 *** (−10.58)	No	-	-	−0.304 *** (−3.19)	0.640	84
		−0.053 *** (−4.57)	Yes	0.076 * (1.76)	−0.393 *** (−4.58)	0.128 (0.94)	0.758	84
$\ln\left(\frac{NO_{x}}{thermal}\right)$	Eastern	−0.091 *** (−9.94)	No	-	-	−5.188 *** (−52.69)	0.628	77
		−0.065 *** (−5.40)	Yes	0.168 *** (3.41)	−0.256 ** (−2.59)	−4.717 *** (−24.53)	0.648	77
	Central	−0.110 *** (−10.04)	No	-	-	−4.893 *** (−69.09)	0.605	84
		−0.098 *** (−6.24)	Yes	−0.021 (−0.43)	−0.144 (−1.30)	−4.700 *** (−31.78)	0.630	84

Note: ***, **, and * represent the statistical significance at the 1%, 5%, and 10% level and the values in the brackets are the *t* statistics.

**Table 6:** Regression outcomes for the ULE reduction effect on smoke dust.

Explained Variable	Region	*DID*	Control Variable	lncar	ln fixedasset	Constant	R^2^ within	Obs.
lnDust	Eastern	−0.032 *** (−2.61)	No	-	-	−1.014 *** (−3.88)	0.097	77
		−0.036 * (−1.94)	Yes	0.196 (0.69)	−0.194 (−0.77)	−1.017 *** (−2.90)	0.092	77
	Central	−0.047 *** (−5.02)	No	-	-	−0.838 *** (−5.78)	0.174	84
		−0.043 *** (−2.93)	Yes	−0.015 (−0.06)	−0.028 (−0.13)	−0.076 *** (−3.60)	0.178	84
$\ln\left(\frac{Dust}{thermal}\right)$	Eastern	−0.043 *** (−3.33)	No	-	-	−5.932 *** (−28.92)	0.143	77
		−0.052 *** (−2.78)	Yes	0.064 (0.23)	−0.063 (0.25)	−6.148 *** (−17.68)	0.154	77
	Central	−0.058 *** (−5.79)	No	-	-	−5.685 *** (−43.30)	0.210	84
		−0.041 *** (−2.66)	Yes	−0.287 (−1.15)	0.124 (0.54)	−5.458 *** (−25.48)	0.220	84

Note: *** and * represent the statistical significance at the 1% and 10% levels and the values in the brackets are the *t* statistics.

**Table 7:** Placebo test results.

Explained Variable	Region	*DID*	Execution Year	Timespan	Control Variable	R^2^ Within	Obs.
$\ln{SO}_{2}$	East	−0.019 * (−1.71)	2011	2009–2014	Yes	0.000	66
	Central	−0.015 * (−1.80)	2011	2009–2014	Yes	0.113	72
lnNOx	East	−0.007 (−1.10)	2012	2011–2014	Yes	0.027	44
	Central	0.001 (0.32)	2012	2011–2014	Yes	0.837	48
lnDust	East	0.009 (0.58)	2013	2011–2014	Yes	0.422	44
	Central	0.014 (0.94)	2013	2011–2014	Yes	0.198	48

Note: * represents the statistical significance at 10% level and the values in the brackets are the *t* statistics.

## Abbreviations

ULE	Ultra-low Emission
DID	Difference-in-Difference
Btu	British Thermal Unit
SO_2_	sulfur dioxide
NO_x_	nitrogen oxide
kWh	kilowatt hour
tce	Ton (Tonne) of Coal Equivalent
Obs	number of observations
Mean	mean value
St.d	standard deviation
Min	minimum value
Median	Median value
Max	maximum value
R^2^ within	goodness of fit within the group
